# Parametric Optimization of Cultural Conditions for Carboxymethyl Cellulase Production Using Pretreated Rice Straw by *Bacillus sp. 313SI* under Stationary and Shaking Conditions

**DOI:** 10.1155/2014/651839

**Published:** 2014-04-29

**Authors:** Varsha Goyal, Arpana Mittal, Anish Kumari Bhuwal, Gulab Singh, Anita Yadav, Neeraj Kumar Aggarwal

**Affiliations:** ^1^Department of Microbiology, Kurukshetra University, Kurukshetra, Haryana 136119, India; ^2^Department of Biotechnology, Kurukshetra University, Kurukshetra, Haryana 136119, India

## Abstract

Carboxymethyl cellulase (CMCase) provides a key opportunity for achieving tremendous benefits of utilizing rice straw as cellulosic biomass. Out of total 80 microbial isolates from different ecological niches one bacterial strain, identified as *Bacillus sp. 313SI*, was selected for CMCase production under stationary as well as shaking conditions of growth. During two-stage pretreatment, rice straw was first treated with 0.5 M KOH to remove lignin followed by treatment with 0.1 N H_2_SO_4_ for removal of hemicellulose. The maximum carboxymethyl cellulase activity of 3.08 U/mL was obtained using 1% (w/v) pretreated rice straw with 1% (v/v) inoculum, pH 8.0 at 35°C after 60 h of growth under stationary conditions, while the same was obtained as 4.15 U/mL using 0.75% (w/v) pretreated substrate with 0.4% (v/v) inoculum, pH 8.0 at 30°C, under shaking conditions of growth for 48 h. For maximum titre of CMCase carboxymethyl cellulose was optimized as the best carbon source under both cultural conditions while ammonium sulphate and ammonium nitrate were optimized as the best nitrogen sources under stationary and shaking conditions, respectively. The present study provides the useful data about the optimized conditions for CMCase production by *Bacillus sp. 313SI* from pretreated rice straw.

## 1. Introduction

Lignocellulosic materials from agriculture and forest management are the largest sources of hexose (C-6) and pentose (C-5) sugars with a potential for the production of biofuels, chemicals, and other economic by-products [1]. Lignocellulosic biomass is mainly composed of plant cell walls, with the structural carbohydrates cellulose and hemicellulose and heterogeneous phenolic polymer lignin as its primary components [2]. The lignocellulosic substrates include woody substrates such as hardwood (birch and aspen, etc.), softwood (spruce and pine, etc.), agroresidues (wheat straw, sugarcane bagasse, corn stover, etc.), dedicated energy crops (switch grass, miscanthus, etc.), weedy materials (*Eicchornia crassipes*,* Lantana camara*, etc.), and municipal solid waste (food and kitchen waste, etc.) [3]. Lignocellulosic biomass is composed of cellulose, hemicellulose, and lignin, as well as other minor components. The recalcitrance of lignocellulosic biomass to enzyme such as the interaction between cellulose and hemicellulose and degree of lignifications necessitates a pretreatment process for increasing its enzymatic digestibility. Pretreatment of biomass plays a critical role in producing materials with acceptable enzymatic digestibility and subsequent fermentability for the production of cellulosic ethanol or other advanced biofuels such as butanol derived from biomass.

Rice straw is an attractive lignocellulosic material for bioethanol production since it is one of the most abundant renewable resources [4] with annual productivity of around 800 million metric tonnes that corresponds with large production of rice straw [5]. For every ton of harvested grain, about 1.35 tons of rice straw remain in the field which generate huge amount of straw annually [6]. Disposal of rice straw is a huge problem as usage of rice straw in biological process, such as composting and biogas production, is limited by slow degradation in bioconverting process [7]. Moreover, it cannot be used as animal feed due to its low digestibility, low protein, and high lignin and silica content [8]. Rice straw is composed of 40% cellulose, 24% hemicellulose, and 25% lignin [9] so it requires a basic step of pretreatment for breakage of lignin and exposure of cellulose and hemicellulose for enzymatic saccharification. Several pretreatment processes including organosolvent [4], ultrasonication [10], alkali [11], steam explosion [12], microwave assisted alkali treatment [13], microwave assisted organic acid treatment [14], hot compress water [15], proton beam radiation [16], ammonia and ionic liquid [17], and acid [18] have been reported for rice straw.

Cellulases are inducible enzymes which are synthesized by microorganisms during their growth on cellulosic materials [19]. The complete enzymatic hydrolysis of cellulosic materials needs different types of cellulase, endoglucanase (1,4-*β*-d-glucan-4-glucanohydrolase; EC 3.2.1.4), exocellobiohydrolase (1,4-*β*-d-glucan glucohydrolase; EC 3.2.1.74), and *β*-glucosidase (*β*-d-glucoside glucohydrolase; EC 3.2.1.21) [20]. Microorganisms are considered to be the main source of cellulases with novel and high specific activities. Microbial sources are the most economic and available sources because microorganisms can grow on inexpensive media such as agriculture and food industries by-products [21]. Various bacteria, actinomycetes, and filamentous fungi produce extracellular cellulases when grown on cellulosic substrates though many actinomycetes have been reported to have less cellulase activity than moulds [22].* Chaetomium*,* Fusarium*,* Trichoderma*,* Penicillium*,and* Aspergillus* are some of the reported fungal species and* Trichonympha, Clostridium, Actinomycetes*,* Bacteroides succinogenes*,* Butyrivibrio fibrisolvens*, and* Ruminococcus albus *are some of the reported bacterial species responsible for cellulosic biomass hydrolysation [23]. Due to increasing demand for energy and the fast depleting petroleum resources there is an increased interest in alternative fuels, especially liquid transportation fuels from lignocelluloses, which led to a new dawn in cellulase research. Various kinds of value added products such as ethanol, organic acids, enzymes, and other chemicals can be made by enzymic hydrolysis of cellulosics; of these processes, ethanol has received the maximum attention as an alternative to gasoline in today's environment [24]. The aim of the present study was to optimize process parameters for alkali assisted acid pretreatment of rice straw for carboxymethyl cellulase enzyme production by* Bacillus sp. 313SI* under stationary and shaking conditions.

## 2. Materials and Methods

### 2.1. Isolation of Bacterial Strain for CMCase Production

Bacterial strains having cellulolytic potential were screened from the soil samples of different niches such as sugarcane field, rice field, paper industry, cattle shed, rotten fruits and vegetables, and samples of cattle dung. Isolation was done by dilution plate method on a carboxymethyl cellulose agar (CMC) medium (Hi media, India) (NaNO_3_-2.0 g/L, K_2_HPO_4_-1.0 g/L, MgSO_4_·7H_2_O-0.5 g/L, KCl-0.5 g/L, CMC-5.0 g/L, Agar-2%, pH 8.0). Screening for cellulolytic activity was followed by visualizing the hydrolysis zone, when the plates were flooded with an aqueous solution of 0.1% Congo red for 15 min and washed with 1 M NaCl [25]. The isolated colonies on these plates were maintained on CMC agar slants at 4°C for further analysis.

### 2.2. Enzyme Assay

Carboxymethyl cellulase activity was assayed by the DNS (3, 5-dinitrosalicylic acid) method [26]. The reaction mixture contained 900 *μ*L of substrate (carboxymethyl cellulose in 10 mM Sodium phosphate buffer pH 7.0) and 100 *μ*L of crude enzyme was incubated at 30°C for 60 min. An appropriate control which contained 100 *μ*L of distilled water instead of crude enzyme extract was also run along with the test. The reaction was terminated by adding 3 mL of 3, 5-dinitrosalicylic acid reagent. The tubes were incubated for 15 min in a boiling water bath for color development and were cooled rapidly. The activity of reaction mixture was measured against a reagent blank at 540 nm. The concentration of glucose released by enzyme was determined by comparing against a standard curve constructed similarly with known concentrations of glucose. One unit of enzyme activity is defined as the amount of enzyme that liberates 1 *μ*g of glucose per minute under the assay conditions.

### 2.3. Pretreatment of Rice Straw

The main components of untreated rice straw were determined to be 38.40% cellulose, 24% hemicelluloses, and 19% lignin using standard procedure [27]. The rice straw was first pretreated with 0.5 M KOH for 4 h at room temperature at the ratio of 1 : 10 for substrate and KOH solution. The pretreated solid was washed with water till neutrality, filtered, and dried. The solid was further treated by 0.1 N H_2_SO_4_ for 1 h at room temperature and then autoclave at 121°C and 15 psi pressure for 15 minutes. The pulp was washed with water till neutrality, filtered, dried, and stored at room temperature for further use.

### 2.4. Optimization of CMCase Production under Stationary and Shaking Conditions

Various physicochemical parameters were analyzed under stationary and shaking conditions for maximum carboxymethyl cellulase enzyme production by* Bacillus sp. 313SI*. The various parameters that were optimized were pretreated rice straw concentration (0.25–2.0% w/v), inoculum concentration (0.25–1.0% v/v), incubation temperature (20–60°C), incubation pH (5.0–9.0), and various additives such as carbon sources (galactose, maltose, carboxymethyl cellulose, starch, mannitol, and cellulose powder) and nitrogen sources (ammonium nitrate, ammonium sulphate, ammonium chloride, beef, tryptone, urea, and potassium nitrate).

### 2.5. Statistical Analysis

The optimization results for different parameters were analyzed using statistical packages system software (SPSS; 16.0). The ANOVA with* post hoc* analysis was applied for within-group comparison. The level of significance was set at 0.05.

## 3. Result and Discussion

### 3.1. Isolation and Identification of Isolated Bacterial Strain

The bacterial strain with maximum carboxymethyl cellulase activity was isolated from cattle shed soil and identified as* Bacillus sp. 313SI* by Xcelris Labs Ltd., Ahmadabad, India, and has been given National Centre for Biotechnology Information (NCBI) accession number JQ734551.1.

### 3.2. Alkali Assisted Acidic Pretreatment of Rice Straw

The effect of alkali assisted acidic pretreatment on chemical composition of rice straw such as cellulose, hemicelluloses, and lignin was analyzed. It was determined that cellulose, hemicelluloses, and lignin content of obtained alkali assisted acidic pretreated rice straw was 59.5%, 8.26%, and 5.17%, respectively. Taherzadeh and Karimi [28] have reported that efficient delignifier should remove a maximum of lignin and minimum of sugars. Pretreatment of lignocelluloses with alkali overcomes the lignin barrier, by dissolving the lignin caused by the breakdown of ether linkages [29]. Lu et al. [30] have examined that hemicelluloses can effectively solubilise and hydrolyze into monomeric sugars and soluble oligomers by dilute sulphuric acid pretreatment. Chandel et al. [31] reported NH_4_OH mediated delignification of sugarcane bagasse which resulted in 41.51% lignin removal as compared to untreated substrate which later improved the enzymatic saccharification of substrate employing commercial cellulase.

### 3.3. Effect of Substrate Concentration

**Figure 1:**
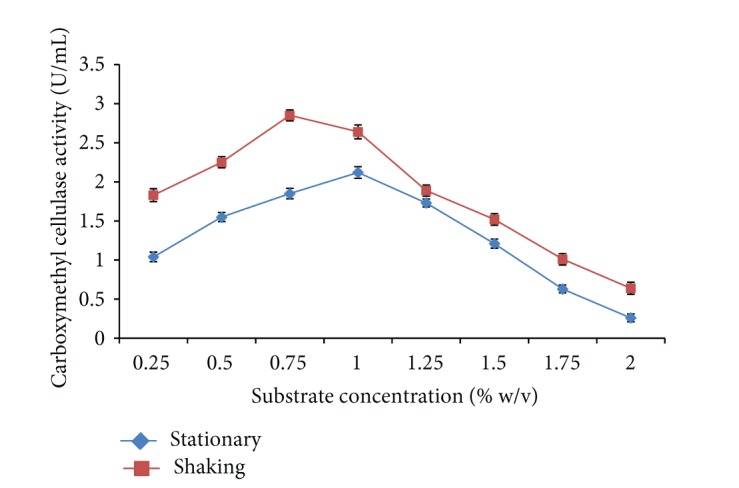
Effect of substrate concentration on carboxymethyl cellulase enzyme production by* Bacillus sp. 313SI* under stationary and shaking conditions of growth. Values in figure are means of three replicates with standard deviation.

Alkali and acid pretreated rice straw was used to analyze the effect of substrate concentration on carboxymethyl cellulase enzyme production by* Bacillus sp. 313SI*. As shown in Figure 1 substrate concentration at 1% w/v and 0.75% w/v was found to be optimized for maximum cellulase activity of 2.12 ± 0.04 U/mL and 2.85 ± 0.06 U/mL under stationary and shaking conditions, respectively. The ANOVA for the data on CMCase as a function of variation due to different concentrations of substrate under stationary conditions (*F* = 327.91; *P* < 0.0001) and shaking conditions (*F* = 325.55; *P* < 0.0001) is statistically significant. Immanuel et al. [32] reported the maximum enzymatic activity with 1.5% pretreated coir fiber.

### 3.4. Inoculum Concentration

**Figure 2:**
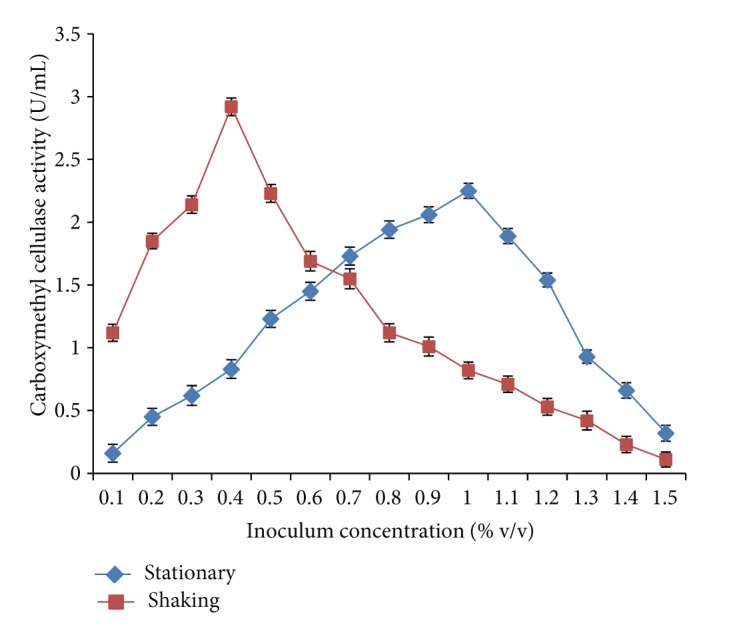
Effect of inoculum concentration on carboxymethyl cellulase enzyme production by* Bacillus sp. 313SI* under stationary and shaking conditions of growth. Values in figure are means of three replicates with standard deviation.

Effective inoculum concentration (0.25–1.0% w/v) for carboxymethyl cellulase enzyme production by* Bacillus sp. 313SI* was evaluated for stationary and shaking conditions. Maximum carboxymethyl cellulase enzyme activity of 2.25 ± 0.06 U/mL was obtained with 1% w/v inoculum concentration under stationary conditions while under shaking conditions 0.4% inoculum concentration had showed the maximum carboxymethyl cellulase enzyme activity of 2.92 ± 0.05 U/mL as shown in Figure 2. The ANOVA for the data on CMCase as a function of variation due to different concentrations of inoculum under stationary conditions (*F* = 475.68; *P* < 0.0001) and shaking conditions (*F* = 584.09; *P* < 0.0001) is statistically significant.* Bacillus subtilis and Bacillus circulans* showed maximum carboxymethyl cellulase enzyme production up to 3% inoculum size [33]. Abou-Taleb et al. [34] also reported that* B. alcalophilus S39* and* B. amyloliquefaciens C2*_*3*_ showed maximum carboxymethyl cellulase enzyme production at 3.0% inoculum size. Das et al. [35] reported optimum inoculum size of 7% for maximum carboxymethyl cellulase enzyme production at 42°C by* Bacillus sp.*

### 3.5. Incubation Time

**Figure 3:**
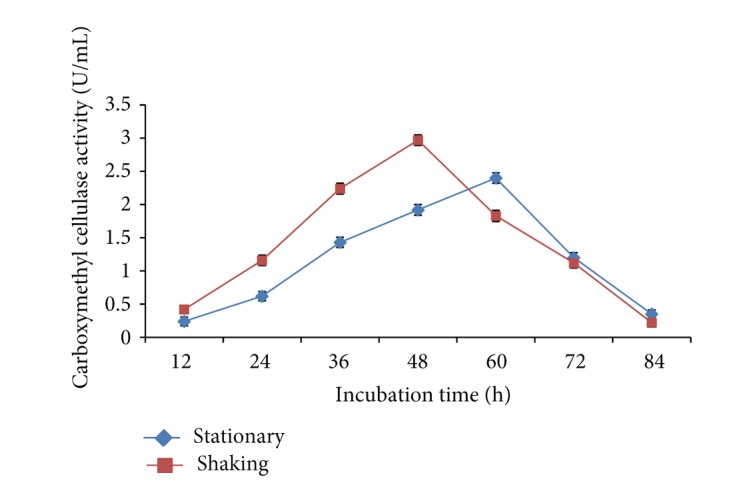
Effect of incubation time on carboxymethyl cellulase enzyme production by* Bacillus sp. 313SI* under stationary and shaking conditions of growth. Values in figure are means of three replicates with standard deviation.

To determine the optimum incubation time for carboxymethyl cellulase enzyme production by* Bacillus sp. 313SI* from pretreated rice straw time course of cultivation was recorded up to 84 h. Carboxymethyl cellulase enzyme production was increased with increase in incubation time and maximum CMCase activity of 2.40 ± 0.07 U/mL was optimized at 60 h under stationary conditions and 2.97 ± 0.06 U/mL at 48 h under shaking conditions as shown in Figure 3. The ANOVA for the data on CMCase as a function of variation due to different time of incubation under stationary conditions (*F* = 890.75; *P* < 0.0001) and shaking conditions (*F* = 947.52; *P* < 0.0001) is statistically significant. Shabeb et al. [36] found maximum carboxymethyl cellulase enzyme activity in* Bacillus subtilis* KO strain after 24 h of incubation period. Heck et al. [37] and Amritkar et al. [38] found maximum carboxymethyl cellulase enzyme activity in* Bacillus spp. B21, Bacillus pumilus,* and* Bacillus subtilis* after 72 h of incubation. Poorna and Prema [39] reported the maximum carboxymethyl cellulase enzyme activity in* Bacillus pumilus* after 120 h of incubation.

### 3.6. Initial pH

**Figure 4:**
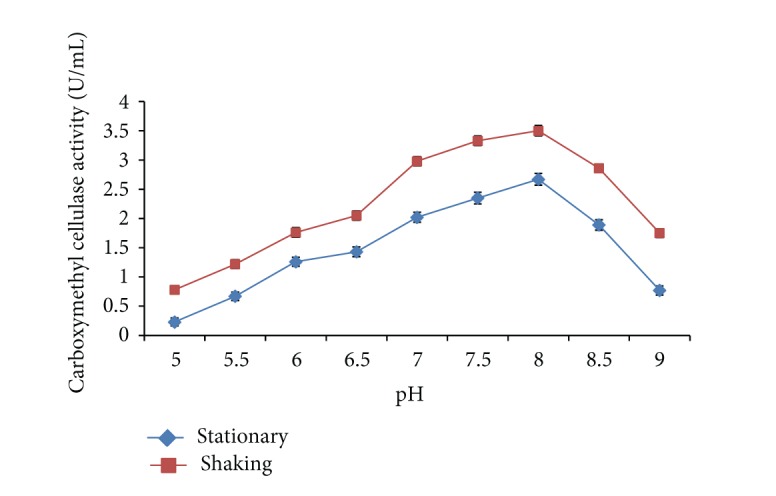
Effect of pH on carboxymethyl cellulase enzyme production by* Bacillus sp. 313SI* under stationary and shaking conditions of growth. Values in figure are means of three replicates with standard deviation.

The initial pH of production medium plays an important role in the production of carboxymethyl cellulase enzyme. The effect of different pH range was optimized on carboxymethyl cellulase enzyme production from* Bacillus sp. 313SI* from pretreated rice straw. Maximum CMCase activity of 2.67 ± 0.06 U/mL was obtained at pH 8.0 under stationary conditions while under shaking conditions this is 3.50 ± 0.07 U/mL as shown in Figure 4. The ANOVA for the data on CMCase as a function of variation due to different pH under stationary conditions (*F* = 627.75; *P* < 0.0001) and shaking conditions (*F* = 741.86; *P* < 0.0001) is statistically significant. These results are in agreement with those of Immanuel et al. [32] who found the cellulolytic enzyme, endoglucanase, obtained from* Cellulomonas*,* Bacillus*, and* Micrococcus spp*. hydrolyzed substrate in the pH range of 4.0 to 9.0, with maximum activity transpiring at pH 7. Ray et al. [33] reported that pH 7–7.5 was more suitable for optimization of cellulase production by* Bacillus subtilis* and* B. circulans*. Gautam et al. [40] found the optimum pH of 7.5 for maximum carboxymethyl cellulase enzyme activity by* Pseudomonas* sp.

### 3.7. Incubation Temperature

**Figure 5:**
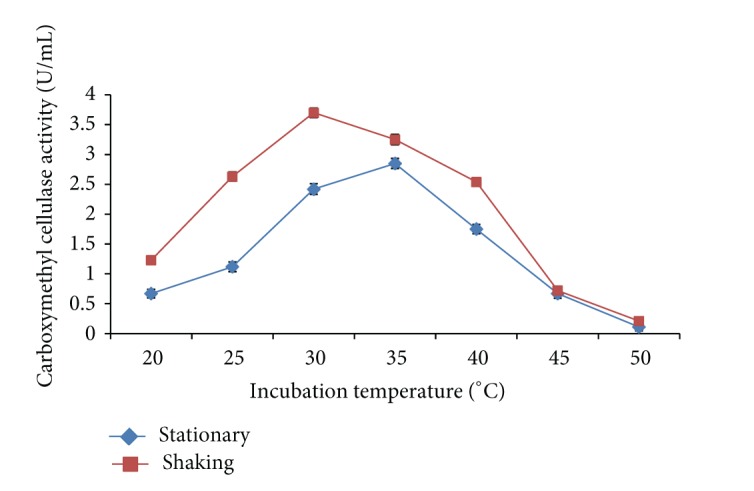
Effect of temperature on carboxymethyl cellulase enzyme production by* Bacillus sp. 313SI* under stationary and shaking conditions of growth. Values in figure are means of three replicates with standard deviation.

The effect of temperature varying between 20°C and 50°C on production of carboxymethyl cellulase enzyme was studied.* Bacillus sp. 313SI* showed maximum carboxymethyl cellulase enzyme activity of 2.85 ± 0.05 U/mL under stationary conditions at 35°C and 3.70 ± 0.06 U/mL under shaking conditions at 30°C as shown in Figure 5. The ANOVA for the data on CMCase as a function of variation due to different temperatures under stationary conditions (*F* = 115.0; *P* < 0.0001) and shaking conditions (*F* = 184.4; *P* < 0.0001) is statistically significant. These results are close to those of Kanmani et al. [21] who found that the carboxymethyl cellulase enzyme produced by* Bacillus pumilis* showed the optimum temperature of 35°C. Rastogi et al. [41] reported two strains DUSELR7 and DUSELR13 as mesophilic carboxymethyl cellulase enzyme producer at 37°C.

### 3.8. Carbon and Nitrogen Sources

**Figure 6:**
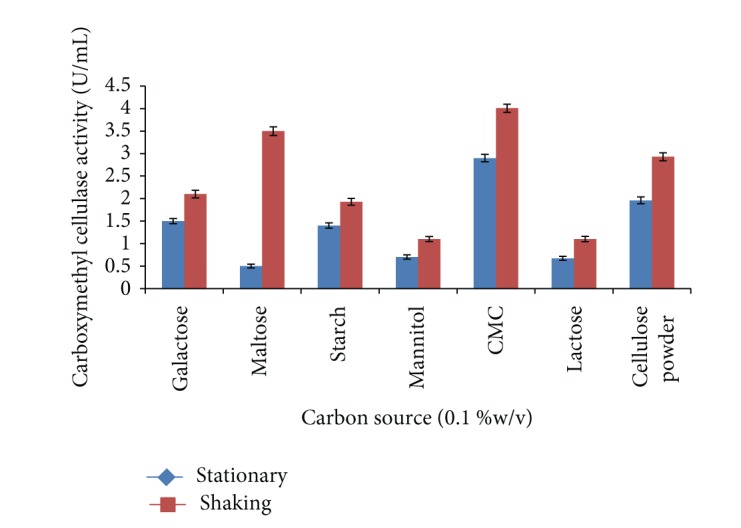
Effect of different carbon sources on carboxymethyl cellulase enzyme production by* Bacillus sp. 313SI* under stationary and shaking conditions of growth. Values in figure are means of three replicates with standard deviation.

Supplementation of carbon and nitrogen sources in medium showed a significant increase in carboxymethyl cellulase enzyme production by* Bacillus sp. 313SI* from pretreated rice straw under stationary and shaking conditions. Effect of different carbon sources (0.1% w/v) on the production of carboxymethyl cellulase enzyme was evaluated as shown in Figure 6.

Carboxymethyl cellulose was optimized as best carbon source for both conditions. The maximum carboxymethyl cellulase enzyme activity of 2.90 ± 0.13 U/mL was recorded under stationary conditions and 4.01 ± 0.06 U/mL was recorded under shaking conditions. The ANOVA for the data on CMCase as a function of variation due to different carbon sources under stationary conditions (*F* = 161.41; *P* < 0.0001) and shaking conditions (*F* = 223.16; *P* < 0.0001) is statistically significant. Carboxymethyl cellulose was most effective as a sole carbon source for carboxymethyl cellulase enzyme production by* Bacillus alcalophilus* S39 [34]. Carboxymethyl cellulose was the best carbon source followed by cellulose for carboxymethyl cellulase enzyme production [42, 43]. 1% (w/v) carboxymethyl cellulose was found to be optimal for carboxymethyl cellulase enzyme production* in Bacillus sp*. [44].

**Figure 7:**
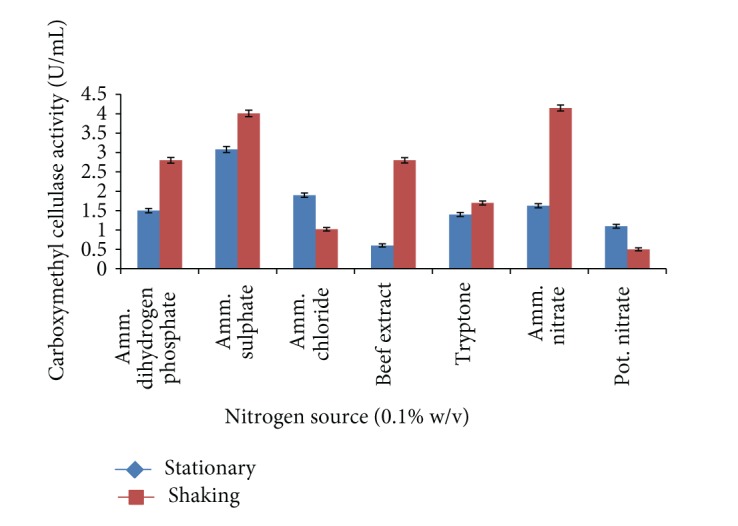
Effect of different nitrogen sources on carboxymethyl cellulase enzyme production by* Bacillus sp. 313SI* under stationary and shaking conditions of growth. Values in figure are means of three replicates with standard deviation.

Similarly the influence of different nitrogen sources (0.1% w/v) on carboxymethyl cellulase enzyme production was evaluated as shown in Figure 7. The results showed that* Bacillus sp. 313SI* gave maximum yield of carboxymethyl cellulase enzyme by added ammonium sulphate, that is, 3.08 ± 0.07 U/mL in medium for stationary conditions and 4.15 ± 0.06 U/mL in medium for shaking conditions. The ANOVA for the data on CMCase as a function of variation due to different nitrogen sources under stationary conditions (*F* = 379.0; *P* < 0.0001) and shaking conditions (*F* = 492.7; *P* < 0.0001) is statistically significant. Balamurugan et al. [45] found that ammonium sulphate and ammonium nitrate are optimum nitrogen sources for carboxymethyl cellulase enzyme in CDB7 and CDB13 isolates at 30°C. Peptone was optimized as the best nitrogen source for carboxymethyl cellulase enzyme production by* Bacillus sp.* at 42°C [35].

## 4. Conclusion

The data gathered in this study provides evidence for CMCase production by* Bacillus sp. 313SI* from alkali assisted acidic pretreated rice straw. Qualitative effect of some carbon and nitrogen sources, incubation time, pH, inoculum concentration, and incubation temperature was studied and optimized for CMCase production by* Bacillus sp. 313SI.* High titre of CMCase production by* Bacillus sp. 313SI* using pretreated cost-effective agroresidue (rice straw) at pH 8.0 and 30°C made it as a potential producer of mesoalkalophilic cellulases which can find wide applications involving saccharification in various lignocellulosic based industries particularly bioethanol industry.
